# The Microbiome in Autoimmune Liver Diseases: Metagenomic and Metabolomic Changes

**DOI:** 10.3389/fphys.2021.715852

**Published:** 2021-10-08

**Authors:** Yanping Zheng, Ying Ran, Hongxia Zhang, Bangmao Wang, Lu Zhou

**Affiliations:** ^1^Department of Gastroenterology and Hepatology, Tianjin Medical University General Hospital, Tianjin, China; ^2^Department of Gastroenterology and Hepatology, Hotan People’s Hospital, Xinjiang, China

**Keywords:** autoimmune liver diseases, microbiome, metagenomic, metabolomic, bile acids

## Abstract

Recent studies have identified the critical role of microbiota in the pathophysiology of autoimmune liver diseases (AILDs), including autoimmune hepatitis (AIH), primary biliary cholangitis (PBC), and primary sclerosing cholangitis (PSC). Metagenomic studies reveal significant decrease of gut bacterial diversity in AILDs. Although profiles of metagenomic vary widely, *Veillonella* is commonly enriched in AIH, PBC, and PSC. Apart from gut microbiome, the oral and bile microbiome seem to be associated with these diseases as well. The functional analysis of metagenomics suggests that metabolic pathways changed in the gut microbiome of the patients. Microbial metabolites, including short-chain fatty acids (SCFAs) and microbial bile acid metabolites, have been shown to modulate innate immunity, adaptive immunity, and inflammation. Taken together, the evidence of host–microbiome interactions and in-depth mechanistic studies needs further accumulation, which will offer more possibilities to clarify the mechanisms of AILDs and provide potential molecular targets for the prevention and treatment in the future.

## Introduction

Autoimmune liver diseases (AILDs) are chronic inflammatory conditions of the liver, including autoimmune hepatitis (AIH), primary biliary cholangitis (PBC), and primary sclerosing cholangitis (PSC) (Biewenga et al., 2020). Poor understanding of etiology makes the diagnosis and treatment of patients with AILDs challenging. In recent years, increasing studies have been focusing on microbiota-host interactions. Imbalanced microbial communities have been suggested to be related with aberrant immune response (Shi et al., 2017). Relationships have been established between the microbiome and autoimmune diseases, such as systemic lupus erythematosus (Katz-Agranov and Zandman-Goddard, 2017), inflammatory bowel disease (Franzosa et al., 2019), and rheumatoid arthritis (Bergot et al., 2019). Particularly, intestinal microbiome and liver could communicate through the biliary tract, portal vein, and systemic circulation, given the special anatomic and physiological relationships of liver and gut. Studies have discovered that liver diseases are intimately linked to the microbial communities of the human gut (Seki and Schnabl, 2012; Miyake and Yamamoto, 2013; LaRusso et al., 2017). Besides bacteria, the involvement of fungus and chlamydia has also been demonstrated in AILDs (Abdulkarim et al., 2004; Wang et al., 2017). Moreover, various metabolites of gut microbiome have been shown to participate in immune development and regulation (Levy et al., 2017).

Since the microbiota plays an important role in the development of the innate and adaptive immune system (Zheng et al., 2020), further study on the interaction between the diseases and microbiota may provide new insights on the etiology and management of AILDs. With the development of high throughput DNA sequencing, the diversity of the human microbiome has been greatly appreciated. The meta-omics approach, which consists of metagenomic, metatranscriptomic, and metabolomic analysis, has allowed for a more comprehensive characterization of the human microbiome (Zhang et al., 2019). Metagenome, a DNA sequencing method, aims to catalog all the genes from the samples (Wang et al., 2015). Metagenome reveals only the microbial composition of the community. The metatranscriptome could record expressed transcripts of the active members under a set of environmental conditions (Shakya et al., 2019). Changes in the composition or function of the gut microbiota lead to metabolite alterations. Through metabolomics, specific bacterial metabolic pathways and metabolites can be defined (Medina-Cleghorn and Nomura, 2014). Apparently, the microbiome is a rising star in the exploration for the prevention, diagnosis, and treatment of AILDs. Thus, we summarize a review of microbiome associated with AILDs from metagenomics and metabolomics, which may be the key for further understanding of the etiology and management of AILDs.

### The Microbiome in Autoimmune Liver Diseases

Metagenomics is a powerful tool that is helpful for the analysis of microbial heterogenicity. It mainly includes two sequencing strategies: amplicon sequencing, most often amplifying portions of the hypervariable regions of 16S rRNA; or shotgun sequencing, which sequences all given genomic DNA from a sample (Rausch et al., 2019). The shotgun metagenomics sequencing can achieve species-level and potentially strain-level of microorganisms (Walsh et al., 2018). In contrast, relative abundances of bacterial taxa derived from the general 16S rRNA is usually defined at the genus level (Walsh et al., 2018).

Studies of the microbiota often focus on the bacterial diversity in the feces of the patients. Collectively, alpha diversity of fecal microbial showed a downward trend in most AILDs patients, which was shown in Table 1 (Sabino et al., 2016; Tang et al., 2018; Wei et al., 2020). However, a German cohort study reported that the alpha-diversity of patients with PSC was similar to controls (Ruhlemann et al., 2019). The microbial communities of human gut were mainly composed of Firmicutes, Bacteroidetes, Actinobacteria, and Proteobacteria at the phylum level (Dekaboruah et al., 2020). Firmicutes and Proteobacteria were increased in both AIH and PBC, while Bacteroidetes showed no significant difference in AIH and PBC when compared to healthy control (HC) (Lv et al., 2020). Remarkably, PSC was characterized by abundant Bacteroidetes and Proteobacteria, whereas Firmicutes were underrepresented (Sabino et al., 2016; Ruhlemann et al., 2019). However, different results can be observed in salivary microbiome and bile microbiome. Statistically significant differences in the phylum level in Firmicutes, Bacteroidetes, and Proteobacteria of the salivary microbiota among the PBC, AIH, and HC groups were not detected (Abe et al., 2018), although the abundance of Bacteroidetes, Firmicutes, and Fusobacteria was also lower in bile microbiome of PSC patients. The abundance of Proteobacteria was higher when compared with patients from the control group (Tyc et al., 2020). These findings suggest that both gut and oral microbiome may be involved in AILDs pathogenesis.

**TABLE 1:** Changes of gut microbiome in autoimmune liver diseases (AILDs).

**Disease**	**Alpha diversity**	**Composition**
AIH	↓	Firmicutes↓ Proteobacteria↑
PBC	↓	Firmicutes↓ Proteobacteria↑
PSC	controversial	Bacteroidetes and Proteobacteria↑ Firmicutes↓

#### The Microbiome in Autoimmune Hepatitis

Microbiome studies are relatively rare in AIH. We only found 6 related articles in PubMed, which are shown in Table 2. Wei et al. (2020) indicated increased abundance in *Veillonella, Klebsiella, Streptococcus*, and *Lactobacillus* in AIH compared to HC. Moreover, they created a model including *Veillonella*, *Lactobacillus*, *Oscillospira*, and *Clostridiales* to distinguish AIH from controls (Wei et al., 2020). *Lachnospiraceae*, *Veillonella*, *Bacteroides*, *Roseburia*, and *Ruminococcaceae* were selected as the AIH microbial biomarkers in another study (Lou et al., 2020). They also reported a higher relative abundance of *Streptococcus* in patients. In Africa, Elsherbiny et al. (2020) reported that *Faecalibacterium, Blautia, Streptococcus, Haemophilus, Bacteroides, Veillonella, Eubacterium, Lachnospiraceae*, and *Butyricicoccus* were enriched in AIH. Given together, most studies confirmed an overrepresentation of *Veillonella* in the gut microbiota of AIH patients. Microbes enriched in gut may aggravate the disease. However, no data support a causal relationship between *Veillonella* and AIH. The actual strains will need to be identified in future studies by shotgun metagenomic sequencing.

**TABLE 2:** Study of microbiome in autoimmune hepatitis (AIH).

**Study**	**Country**	**Sample**	**Groups**	**AIH-enriched taxa**	**Controls-enriched taxa**
Lou et al., 2020	China	Stool	AIH(37) vs. HC(48)	15 genera such as *Veillonella*, *Faecalibacterium*, *Klebsiella*, *Akkermansia*, *Enterobacteriaceae_unclassified*, *Megasphaera*, and so on	19 genera such as *Pseudobutyrivibrio*, *Blautia*, *Lachnospira*, *Erysipelotrichaceae_incertae_sedis*, *Ruminococcaceae_incertae_sedis*, *Phascolarctobacterium*, and *Alistipes* and so on
Wei et al., 2020	China	Stool	AIH(91) vs. HC(98)	*Veillonella*, *Klebsiella*, *Streptococcus*, and *Lactobacillus*	*Clostridiales, RF39, Ruminococcaceae*, *Rikenellaceae, Oscillospira, Parabacteroides*, and *Coprococcus*
Lin et al., 2015	China	Stool	AIH (24) vs. HC(8)	/	*Bifidobacterium, Lactobacillus*
Elsherbiny et al., 2020	Egypt	Stool	AIH(15) vs. HC(10)	*Faecalibacterium, Blautia, Streptococcus*, *Haemophilus, Bacteroides, Veillonella*, *Eubacterium, Lachnospiraceae*, and *Butyricicoccus*	*Prevotella, Parabacteroides*, and *Dilaster*
Abe et al., 2018	Japan	Stool	AIH(17) vs. HC(15)	*Lactobacillales*	*Clostridium subcluster XIVa*
Lv et al., 2020	Germany	Stool	AIH(72) vs. HC(95)	*Veillonella*, facultative anaerobic genera *Streptococcus* and *Lactobacillus*	*Bifidobacterium, Faecalibacterium*
Abe et al., 2018	Japan	Saliva	AIH(17) vs. HC(15)	*Veillonella*	*Streptococcus, Fusobacterium*

Probiotics were believed to restore the composition of the gut microbiome (Hemarajata and Versalovic, 2013). It can also participate in regulating the immune system (Liu et al., 2018). *Bifidobacteria*-based probiotics have been shown to confer health benefits on the host by regulating gut microbiota (O’Callaghan and van Sinderen, 2016). There is a depletion of *Bifidobacterium* in AIH (Lin et al., 2015; Lv et al., 2020). Furthermore, patients with lower *Bifidobacterium* failed to achieve remission (Lv et al., 2020). Zhang et al. (2020) reported that *Bifidobacterium lactis 420* have beneficial functions in alleviating experimental autoimmune hepatitis. It suggested probiotics supplements may help to treat AIH in the future.

Except for gut microbiota, it is increasingly recognized that the oral cavity microbiota could also affect the host health (Arweiler and Netuschil, 2016). Dysbiosis of the oral microbiota has been found to be related to the pathogenesis of autoimmune diseases, such as inflammatory bowel diseases and systemic lupus erythematosus (van der Meulen et al., 2019; Elmaghrawy et al., 2020). There is a significant increase in *Veillonella* in the oral microbiota of AIH patients when compared with the HC, whereas *Streptococcus* is decreased (Abe et al., 2018). However, *Veillonella* is closely related to oral infectious diseases (Luo et al., 2020), which may influence the result. More research is needed. Notably, the change of *Veillonella* in oral bacterial community is consistent with that of fecal in AIH patients, although the relationship between the gut microbiota and the oral microbiota is still unknown. We can look forward to seeing that *Veillonella* strain may become a microbial marker in AIH.

#### The Microbiome in Primary Biliary Cholangitis

Primary biliary cholangitis is a chronic occult disease which can progress to cirrhosis, and ultimately to liver failure and even death (Hirschfield et al., 2018). Lammert et al. (2021) found that fecal microbiota were related to the fibrosis and cirrhosis of PBC. Therefore, the analysis of microbiota composition in patients with PBC is meaningful. A study containing 60 UDCA treatment naive PBC patients and 80 healthy controls found 12 bacteria (Table 3) whose abundance changed in PBC compared with HC in China. *Haemophilus*, *Veillonella*, *Clostridium*, *Lactobacillus*, *Streptococcus*, *Pseudomonas*, *Klebsiella*, and an unknown genus in the family of *Enterobacteriaceae* were increased, and Bacteroidetes spp., *Sutterella*, *Oscillospira*, *Faecalibacterium* were decreased in PBC (Tang et al., 2018). These altered genera can help to discriminate PBC with HC (Tang et al., 2018). The relative abundance of *Streptococcus* was reported to be positively correlated with AST in alcoholic liver disease (Zhong et al., 2021). Regrettably, its role in PBC needs to be explored. Further, later study has reported that a decrease abundance of *Faecalibacterium* was associated with treatment non-responders, suggesting that *Faecalibacterium* may be beneficial for treatment response in patients with PBC (Furukawa et al., 2020). Another study from China also indicated more abundant genera including *Haemophilus*, *Veillonella*, *Lactobacillus*, *Streptococcus*, and *Klebsiella* in PBC (Lv et al., 2016). Besides *Streptococcus* and *Lactobacillus*, *Bifidobacterium* was proved to be more abundant in the PBC group compared with healthy control in Japanese (Furukawa et al., 2020). In addition, this study observed a significant reduction in the diversity of *Clostridiales*, which included amounts of butyric acid-producing symbiotic bacteria (Furukawa et al., 2020), although the findings above showed gut microbiota was strongly related to PBC. Evidence above also revealed that the change of microbial abundance is not just limited to one specie. This makes it more difficult to understand the potential mechanism between microbiome and disease. Present studies of metagenomics of gut microbiota are all from Asian patients and thus have certain limitations. In the future, more metagenomics studies of PBC are needed to identify the possible pathogens.

**TABLE 3:** Study of the microbiome in primary biliary cholangitis (PBC).

**Study**	**Country**	**Sample**	**Groups**	**PBC-enriched taxa**	**HC-enriched taxa**
Tang et al., 2018	China	Stool	PBC(60) vs. HC(80)	*Haemophilus, Veillonella, Clostridium, Lactobacillus*, *Streptococcus, Pseudomonas, Klebsiella*, *Enterobacteriaceae*	*Bacteroidetes* spp., *Sutterella, Oscillospira*, *Faecalibacterium*
Furukawa et al., 2020	Japan	Stool	PBC(76) vs. HC(23)	*Bifidobacterium, Streptococcus, Lactobacillus*, *Enterococcus*	*Lachnospiraceae*, *Ruminococcaceae* of class *Clostridia*
Abe et al., 2018	Japan	Stool	PBC(39) vs. HC(15)	*Lactobacillales*	*Clostridium subcluster XIVa*
Lv et al., 2016	China	Stool	PBC(42) vs. HC(30)	*γ-Proteobacteria, Enterobacteriaceae*, *Neisseriaceae, Spirochaetaceae, Veillonella*, *Streptococcus, Klebsiella, Actinobacillus pleuropneumoniae, Anaeroglobus geminatus*, *Enterobacter asburiae, Haemophilus parainfluenzae, Megasphaera micronuciformis*, *Paraprevotella clara*	*Acidobacteria, Lachnobacterium* sp., *Bacteroides eggerthii, Ruminococcus bromii*
Abe et al., 2018	Japan	saliva	PBC(39) vs. HC(15)	*Veillonella, Eubacterium*	*Fusobacterium*

Gut microbiome is the hotspot in PBC research, whereas the investigation of microbiology at other body sites is also going on. As we all know, the biliary tract is traditionally considered sterile or has few bacteria. Previous study has reported that there are bacteria in PBC patients’ bile, mainly including *Staphylococcus aureus, Enterococcus faecium*, and *Streptococcus pneumoniae* (Hiramatsu et al., 2000). Additionally, it is worth noting that *Propionibacterium acnes* 16S rRNA gene has been detected in epithelioid granuloma of PBC livers, but less in adjacent hepatic parenchyma (Harada et al., 2001). *Propionibacterium acnes* has been suggested as a most likely infectious pathogen of sarcoidosis, a kind of autoimmune disease (Yamaguchi et al., 2021). This study indicated that *Propionibacterium acnes* may also play a role in PBC which required further investigation. Dysbiosis of the oral microbiome has also been defined in PBC. It was characterized by increased relative abundances in *Eubacterium* and *Veillonella* as well as decreased abundances in *Fusobacterium* (Abe et al., 2018). Above all, *Veillonella* is consistently overrepresented in both stool and saliva of patients with PBC, indicating that *Veillonella* is closely associated with PBC. Further studies are warranted to investigate how *Veillonella* interact with PBC.

#### The Microbiome in Primary Sclerosing Cholangitis

The 16S rRNA gene analysis has also been a major method of bacterial analysis in PSC. Studies of the changes of microbiome in PSC were shown in Table 4. At present, there was only one study that has measured genetic diversity of fecal microbiota by shotgun metagenomic sequencing. It demonstrated the microbial gene richness reduced markedly in patients with PSC compared with HC (Kummen et al., 2020). Nine species showed an increased prevalence and 5 species were less prevalent in PSC compared to HC (Kummen et al., 2020). This study suggested that *Veillonella atypica*, *Veillonella parvula*, and an unclassified *Veillonella* species were more prevalent in PSC patients (Kummen et al., 2020). There is no study illustrating the effect of specific *Veillonella* strain in PSC. The relative abundance of *Veillonella* genera were also increased in children and teenagers with PSC (Cortez et al., 2020). Intriguingly, it has been proved that the abundance of *Veillonella* decreased after effective treatment of UDCA (Kummen and Hov, 2019). Evidence above suggested that the abundance of *Veillonella* was closely related to PSC, but it is not sufficient to distinguish PSC and controls (Ruhlemann et al., 2017). Then Ruhlemann et al. (2017) established a diagnosis model consisting of *Veillonella*, *Clostridiales*, *Lachnospiraceae*, and *Coprococcus* to help to differentiate PSC from HC.

**TABLE 4:** Study of the microbiome in primary sclerosing cholangitis (PSC).

**Study**	**Country**	**Sample**	**Groups**	**PSC-enriched taxa**	**HC-enriched taxa**
Lapidot et al., 2021	Israel	Stool	PSC(35) vs. HC(30)	32 species including *Clostridium XlVa*, *Clostridium symbiosum, Clostridium perfringens, Streptococcus salivarius*, *Veillonella dispar, Ruminococcus gnavus, Bacteroides fragilis*, *Enterobacteriaceae, Lactobacillus*, and *Blautia*	261 species including *Bacteroides thetaiotaomicron* and *Faecalibacterium prausnitzii*
Kummen and Hov, 2019	Norway	Stool	PSC(85) vs. HC(263)	*Veillonella*	12 genera including *ML615J-28,Succinivibrion*, *Desulfovibrio, RF32*, *Phascolarctobacterium, Coprococcus*, and so on
Cortez et al., 2020	Brazil	Stool	PSC(11) vs. HC(23)	*Streptococcus, Veillonella*	/
Vaughn et al., 2019	United States	Stool	PSC/IBD(7) vs. HC(8)	*Megamonas*	*Clostridium XIVa, Faecalibacterium*
Ruhlemann et al., 2019	Germany and Norway	Stool	PSC(73) vs. HC(98)	p.Proteobacteria, *g.Parabacteroides*, *bacteroides* spp., c.Gammaproteobacteria, *g.Streptococcus, c.Bacilli*, *o.Lactobacillales, g.Veillonella*	*Coprococcus* spp.
Sabino et al., 2016	Belgium	Stool	PSC&PSC-IBD(52) vs. HC(52)	*Enterococcus, Streptococcus*, *Lactobacillus* and *Fusobacterium*	/
Kummen et al., 2020	Germany and Norway	Stool	PSC(136) vs. HC(158)	*Clostridium clostridioforme*, *Clostridiales bacterium17 47FAA*, *Clostridium bolteae, Bifidobacterium bifidum, Clostridium symbiosum*, *Eggerthella lenta, Escherichia unclassified, Eggerthella unclassified*, *Clostridium citroniae, Veillonella atypica*, *Veillonella parvula*, and *an unclassified Veillonella species*	*Coprobacter fastidiosus, Alistipes senegalensis, Eubacterium ramulus*, *Eubacterium hallii, Lachnospiraceae* *bacterium 71 58FAA*
Bajer et al., 2017	Czechia	Stool	PSC(43) vs. HC(31)	*Rothia, Enterococcus, Streptococcus*, *Clostridium, Veillonella*, and *Haemophilus*	*Coprococcus*
Liwinski et al., 2020	Germany	Bile	PSC(43) vs. HC(22)	*Enterococcus faecalis, Staphylococcus epidermidis, Streptococcus sanguinis*, *Enhydrobacter aerosaccus, Prevotella pallens, Veillonella dispar*	*Gemella sanguinis, Streptococcus* *gordonii*
Pereira et al., 2017	Finland	Bile	PSC(80) vs. HC(46)	/	An unclassified *Enterobacteriaceae*, *Neisseria*, *Campylobacter*, an unclassified *Neisseriaceae*
Lapidot et al., 2021	Israel	Saliva	PSC(35) vs. HC(30)	*Streptococcus salivarius, Prevotella histicola, Rothia mucilaginosa*, *Veillonella parvula, Actinomyces*, *Campylobacter concisus*, *Bifidobacterium stellenboschense*, *Bacteroidales genus Phocaeicola*	/

To our knowledge, 70% of PSC patients have underlying inflammatory bowel disease (IBD) (Weismuller et al., 2017). Thus, it is necessary to distinguish the microbial profile of PSC with or without IBD. Published data suggested that the fecal microbiota of patients with PSC was significantly different from both HC and patients with IBD (Ruhlemann et al., 2019). At the genus level, *Rothia*, *Lactobacillus*, *Streptococcus*, and *Veillonella* were observed overrepresented specifically in PSC patients (Bajer et al., 2017). *Coprobacillus*, *Escherichia*, *Corynebacterium*, and *Lactobacillus* genera were related to PSC-IBD, but not PSC without IBD (Bajer et al., 2017). However, *Rothia*, *Streptococcus*, *Enterococcus*, *Veillonella*, *Clostridium*, and *Haemophilus* were more abundant in all subgroups of PSC (Bajer et al., 2017). Until now, studies have shown that all the treatments can’t change the natural history of PSC. Evaluating the difference of microbiota between PSC with or without IBD may help to find specific treatments for different subgroups of PSC. A randomized placebo-controlled crossover study including 14 PSC patients suggested that probiotics supplement didn’t alter the symptoms, liver biochemistry, or liver function in PSC (Vleggaar et al., 2008). However, evidence showed that fecal microbiota transplantation could increase bacterial diversity and was related with decreased alkaline phosphatase in patients with PSC (Allegretti et al., 2019).

Currently, composition of the bile microbiome in PSC is gradually emerging. In previous studies of bile microbiome in PSC, an over-representation of *Enterococcus* spp., *Prevotella* spp., *Staphylococcus* spp., *Lawsonella* spp., *Veillonella dispar*, and *Cutibacterium* was observed (Liwinski et al., 2020). *Klebsiella* spp. was also found in bile fluid of PSC patient (Liwinski et al., 2020). It has been reported that *Klebsiella pneumoniae* is associated with intestinal barrier dysfunction (Nakamoto et al., 2019). Nevertheless, the relationship between *Klebsiella* and PSC could not be confirmed in a cohort including 62 patients (Ruhlemann et al., 2019). On the contrary, Pereira et al. (2017) found similar bacterial communities of bile in non-PSC subjects and early stage PSC patients, which indicated that the initiation of PSC may not be associated with alteration in bile microbial communities.

The characteristic of oral microbiota has also been defined in PSC. Alpha-diversity of the salivary microbiome was not changed when comparing PSC with HC, but there is an overrepresentation of *Streptococcus salivarius, Prevotella histicola, Rothia mucilaginosa, V. parvula, Actinomyces, Campylobacter concisus, Bifidobacterium stellenboschense*, and Bacteroidales genus *Phocaeicola* (Lapidot et al., 2021). Furthermore, *S. salivarius, V. parvula, Actinomyces*, and *Bifidobacterium* were both significantly enriched in both the saliva and the fecal samples in patients with PSC compared with HC (Lapidot et al., 2021). Combining the analyses of fecal and oral microbiota studies may help to find out the specific bacterium which participates in the pathogenesis of disease.

#### Specific Microbiome in Autoimmune Liver Diseases

As we all know, Helicobacter mainly colonizes in stomach. Helicobacter species may also partly participate in the pathogenesis of AILDs. In a previous study, Helicobacter species have been detected in livers from adults suffering from AIH, PSC, and PBC using PCR or DNA sequencing (Nilsson et al., 2000; Casswall et al., 2010). However, this study could not decide whether the Helicobacter specie play a pathogenetic role in AILDs because of the lack of healthy controls. Boomkens et al. (2005) in the Netherlands demonstrated that Helicobacter species do not play a causal role in the pathogenesis of the PBC and PSC, by comparing Helicobacter species-specific DNA in liver tissue of patients with PBC/PSC and a control group. But another study involving 25 patients with end-stage PSC and 31 controls suggests a contributory role of *Helicobacter pylori* in the pathogenesis of PSC (Krasinskas et al., 2007). *H. pylori* gene can be detected in liver tissue samples of patients with PBC (Krasinskas et al., 2007), whereas there is no correlation between *H. pylori* antibody and PBC (Durazzo et al., 2004). Cross-reaction can be observed between *H. pylori* and mitochondria antibody of the bile duct cells, although there is no evidence so far of a common epitope between *H. pylori* and bile duct cell (Bogdanos et al., 2004). In general, the correlation between *H. pylori* antibody and AILDs is still controversial.

In addition to bacteria, the role of chlamydia in AILDs has also been studied. Although Chlamydia pneumoniae specific 16S rRNA gene and antigen can be found in PBC liver (Leung et al., 2003). No difference of Chlamydia pneumoniae IgG was seen in PBC patients compared to post-hepatitis cirrhosis patients, suggesting that infection with chlamydia may not be the triggering agent of PBC (Liu et al., 2005). Recently, Lemoinne et al. (2020) found that the fungal microbiota of patients with PSC displayed an increased biodiversity. Moreover, their study suggested that PSC was associated with the increase of *Exophiala* genus and *Sordariomycetes* class, with a decrease of the *Saccharomycetales* order, *Saccharomycetes* class, *Saccharomycetaceae* family, and *S. cerevisiae* species (Lemoinne et al., 2020). Investigation of other microbiome such as chlamydia and fungi may provide a new direction for microbiome study in the future.

It is urgent to find related bacteria of AILDs, because of difficult diagnosis at the early stage in disease. Cumulative evidences show a linkage between microbiome and AILDs. The genera of *Veillonella* is predominant in the gut of AIH, PSC, and PBC patients, showing a vital role in AILDs. However, little is known about the potential mechanism of it in AILDs. The present result showed *Veillonella* may be enriched by suppression of bile acid synthesis (Loomba et al., 2021). Exploring the potential mechanism of microbiome in AILDs could be an opportunity for disease diagnosing and treating.

### Functional Analyses of the Microbiome in Autoimmune Liver Diseases

**FIGURE 1:**
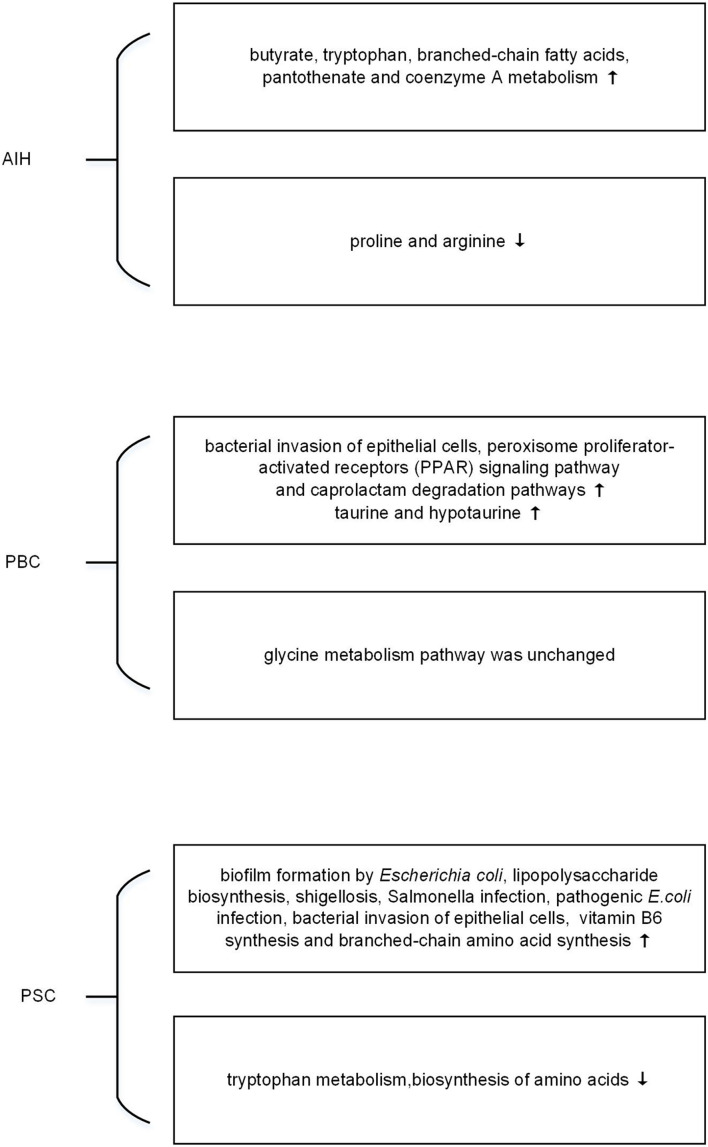
The changes of microbial function predicted by metagenomic in autoimmune liver diseases (AILDs).

Exploring the response of microbiome may help to find the induction factor of AILDs. Metagenomics is limited to reveal the functional activities of microorganisms, while metatranscriptomics is applied to explore the rapid response and expressed biological signatures of microorganisms to the external stimuli (Franzosa et al., 2014; Ram-Mohan and Meyer, 2020). However, there is still no metatranscriptomics research in AILDs. By PICRUSt, a tool used to infer the functional profile of microbial community, the microbial function is shown in Figure 1. Elsherbiny et al. (2020) demonstrated that butyrate, tryptophan, branched-chain fatty acids, pantothenate, and coenzyme A metabolisms were improved in microbial communities of AIH. However, the metabolism associated with proline and arginine was reduced (Elsherbiny et al., 2020). Changes in the metabolites have been verified in metabolomics, which will be illustrated later. Furthermore, bacterial invasion of epithelial cells, peroxisome proliferator-activated receptors (PPAR) signaling pathway, and caprolactam degradation pathways were enriched in PBC (Tang et al., 2018). The selective destruction of biliary epithelial cells is the key step in the pathogenesis of PBC (Selmi et al., 2010). PPAR agonists is suggested to regulate bile acid pool, and reduce inflammation and fibrosis of liver (Gerussi et al., 2020). Intriguingly, UDCA use not only influences the relative abundance of microbial species in feces but also alters the metabolic pathways of microbiota. The metabolic pathway predicted by 16s rRNA sequencing data showed elevation in taurine and hypotaurine metabolism in PBC after UDCA treatment, whereas glycine metabolism pathway had no difference with that of UDCA-naive PBC (Chen et al., 2020). The change of bile acids in PBC has been confirmed by metabolomic study (Yang and Duan, 2016). As for PSC patients, evidence showed that inferred microbiome functions were significantly different between PSC and healthy controls. There were an increase of ‘biofilm formation by *Escherichia coli*,’ ‘lipopolysaccharide biosynthesis,’ ‘shigellosis,’ ‘Salmonella infection,’ ‘pathogenic *E. coli* infection’ and ‘bacterial invasion of epithelial cells,’ and a decrease of ‘tryptophan metabolism,’ ‘biosynthesis of amino acids’ in PSC (Liwinski et al., 2020). In addition, Kummen et al. (2020) demonstrated that patients with PSC had more metabolic pathways related to vitamin B6 synthesis and branched-chain amino acid synthesis compared to healthy controls. In short, pioneering work has indicated that metatranscriptomics had their functional potential in AILDs. Further study is needed in the field.

### Microbial Metabolites Associated With Autoimmune Liver Diseases

Microbial metabolites are emerging to be important effectors mediating the impact of microbiota on host immune responses and are critical for host-microbiota interactions. The gut microbial metabolites contain a wide variety of molecules ranging from short-chain fatty acids (SCFAs) and vitamins to secondary bile acids and neurotransmitters (Kummen and Hov, 2019). Colonic microbiota can transform carbohydrates into SCFAs, including acetate, propionate, and butyrate (Topping and Clifton, 2001). SCFAs have been verified to participate in regulating both innate immunity and antigen-specific adaptive immunity (Kim, 2018). It cannot only suppress the proinflammatory activation of macrophage but also promote peripheral regulatory T-cell generation (Dohmen et al., 2002; Arpaia et al., 2013). Previous study indicated that SCFAs can be uptaken by liver (Cummings et al., 1987). Recently, Zhang et al. (2020) demonstrated that the fecal butyrate was decreased in AIH patients compared to HC. The cause of butyrate reduction in AIH is still unknown. Furukawa et al. (2020) demonstrated a significant reduction in the diversity of the order *Clostridiales*, which included butyric acid-producing bacteria. It may partly explain the reduction of butyrate. In addition, SFCAs supplementation has been proved to ameliorate experimental autoimmune hepatitis (Hu et al., 2018). Furthermore, Wu et al. (2017) have shown that butyrate could ameliorate experimental autoimmune hepatitis through maintaining the integrity of small intestine via inhibiting TLR4 signaling pathway. Altogether, SCFAs may have beneficial effects on liver health through various mechanisms.

Excessive intrahepatic accumulation of bile acids can aggravate liver injury in cholestatic diseases (Schmucker et al., 1990). Bile acids not only act as a stimulator of numerous inflammatory mediators but also induct mitochondrial reactive oxygen species, which may contribute to the progression of cholestatic liver diseases (Li et al., 2017). In addition, bile acids are crucial for the immune modulation and study has demonstrated bile acids metabolites play a pivotal role to regulate the balance of TH17 and Treg cells (Hang et al., 2019). Bile acid can regulate gut microbial composition and immune response (Ramirez-Perez et al., 2017). On the contrary, the microbiome also plays a central role in bile acid homeostasis. Primary bile acids are synthesized in the liver and metabolized into secondary bile acids by microbiota in the gut (Ridlon and Bajaj, 2015). Liwinski et al. (2020) detected decreased concentrations of most bile acids in bile of PSC patients, except for the secondary bile acid-taurolithocholic acid. Torres et al. (2018) also found PSC-IBD patients had a significant decrease in total stool bile acid pool compared to HC. Chen et al. (2020) identified decreased levels of lithocholic acid, glycodeoxycholic acid, and increased levels of cholic acid, taurochenodeoxycholic acid, chenodeoxycholic acid, and taurocholic acid in feces of PBC. UDCA, a kind of secondary bile acid, is just a small fraction of total bile acids. So far, it’s the main therapeutic drug for PBC, which has been proved to significantly improve the outcomes of patients with PBC (Santiago et al., 2018). The production of UDCA can be modified by intestinal bacteria in gut (Tonin and Arends, 2018). UDCA was absent in germ-free mice (Tabibian et al., 2016). Farnesoid X receptor, which plays a pivotal role in regulating liver inflammation and the extent of inflammatory responses, can be activated by bile acids (Ding et al., 2015). Oral vancomycin significantly reduced the concentration of secondary fecal bile acids, emphasizing the role of microbiota in transformation of primary bile acid (Vaughn et al., 2019). It’s worth noting that the abundance of *Veillonella* and *Klebsiella* have been proved to be negatively correlated with the level of secondary bile acids in serum (Chen et al., 2020).

As mentioned above, evidence showed the change of microbiota metabolomics was associated with AILDs. But there is much of the gut metabolome that remains uncharacterized. Untargeted metabolomics has great potential to identify novel molecules in AILDs in the future.

## The Role of Microbiome in the Pathogenesis of Autoimmune Liver Diseases

**FIGURE 2:**
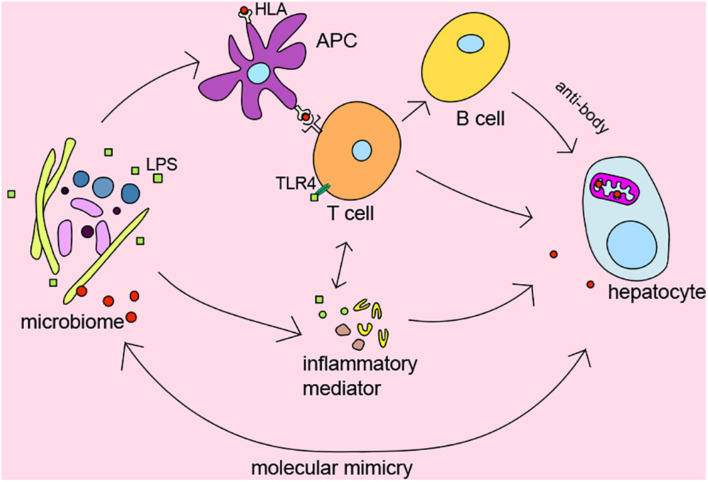
The role of microbiome in pathogenesis of AILDs.

It’s widely recognized that genetic predisposition in combination with exposure to environmental triggers and immunity dysregulation play a vital role in the pathogenesis of AILDs (Arndtz and Hirschfield, 2016). We summarized the role of intestinal microbiota in AILDs pathogenesis as follows in Figure 2.

Several genome-wide association studies indicated that there is significant association between HLA DR3, DR4, and AIH (de Boer et al., 2014; van Gerven et al., 2015), HLA DRB1^∗^08, and PBC (Invernizzi et al., 2005). Moreover, early studies from Norway and the United Kingdom also identified HLA-DR3 (*DRB1^∗^0301*) as the susceptible gene of PSC (Schrumpf et al., 1982). HLA gene has been shown to affect the microbial composition of the late infant gut in a cohort study from southeast Sweden (Russell et al., 2019). The microbiome participates in regulating inflammatory and immune responses as trigger factors. However, the detailed relationship between HLA and microbiome in AILDs still needs further exploration.

To our knowledge, T lymphocyte plays a central role in the immunopathogenesis of AILDs (Ishibashi et al., 2003; Ichiki et al., 2005; Henriksen et al., 2017). Interestingly, Clostridium has been reported to regulate the induction of T regulatory cells by providing bacterial antigens and SCFAs (Atarashi et al., 2013). Bacterial metabolites SCFAs have been demonstrated to affect the activity of T regulatory cells and reduce the levels of pro-inflammatory cytokines such as IFN-γ, IL-6, IL-1b, MIP-2, and TNF-α (Felice et al., 2015; Bhaskaran et al., 2018). In addition, bile acid metabolites, which mainly regulated by microbiota, were reported to inhibit TH17 cells by binding to the key transcription factor retinoid-related orphan receptor-γ and regulate T regulatory cells through the production of mitochondrial reactive oxygen species (Hang et al., 2019).

It is currently believed that chronic bacterial infection might play a part in the initiation or development of the autoimmune status in patients with AILDs (Haruta et al., 2010). Many studies suggest the alteration of microbes in AILDs, but the causality of this relationship is unclear. In fact, additional intestinal barrier dysfunction has been proved to exacerbate liver injury in mice (Zhang et al., 2021). Bacteria and endotoxin would enter the systemic circulation and trigger immune response because of intestinal barrier dysfunction (Krentz and Allen, 2017). Recently, it has been reported that translocation of *Enterococcus gallinarum* to the liver in germ-free C57BL/6 mice could trigger autoimmune responses (Manfredo Vieira et al., 2018). Zhang et al. (2020) found that *B. lactis 420* mitigated experimental autoimmune hepatitis through regulating intestinal barrier and liver immune cells (macrophage and Th17 cells). Specifically, the NOD.c3c4, which could spontaneously develop biliary inflammation, is a model of the human biliary disease primary biliary cirrhosis (Koarada et al., 2004). Biliary inflammation is ameliorated in antibiotic-treatment and germ-free NOD.c3c4 mice (Schrumpf et al., 2017). Nakamoto et al. (2019) demonstrated that gnotobiotic mice inoculated with PSC-derived microbiota was more susceptible to hepatobiliary injuries. Previous evidence shown that microbiome may be the promoting factors of AILDs. Antibacterial treatment may be effective options to suppress the development of the disease.

Molecular mimicry between immunodominant epitopes of the pathogen and self-peptides has been hypothesized to be the key event leading to the disease. It may provide clues to explain the relationship between microbes and disease. Investigations have detected that PDC-E2 of *E. coli* is molecularly similar to human PDC-E2, the immunodominant target of AMAs in PBC (Shimoda et al., 1995). Meanwhile, PDC-E2-like proteins of *Novosphingobium aromaticivorans* were more similar with human PDC-E2 than that of *E. coli* (Kaplan, 2004). Although *N. aromaticivorans* hasn’t been detected in the liver of patients with PBC (Tanaka et al., 1999), a study has reported that *N. aromaticivorans* is present in approximately 25% of fecal samples from patients and controls (Selmi et al., 2003). *Lactobacillus delbrueckii* and *Mycobacterium gordonae* might also induce loss of tolerance to human mitochondrial proteins in genetically susceptible individuals due to molecular mimicry and immunological cross-reactivity (Vilagut et al., 1994, 1997; Bogdanos et al., 2008). Recent findings also suggested that antibodies against *Yersinia enterocolitica* were present in PBC patients (Yamaguchi et al., 1994). Interestingly, Roesler et al. (2003) identified β-subunit of bacterial RNA-polymerase, a non-species-specific bacterial protein, as the target of antibodies in PBC. Besides bacterium, mycoplasma has also been suggested as a causative factor in the etiopathogenesis of PBC via ‘molecular mimicry’ (Berg et al., 2009). It is worth noting that PDC-E2 is highly conserved from prokaryotes to advanced organisms, which may be the reason why multiple microorganisms are related to AILDs. These results confirmed the hypothesis that autoimmunity in AILDs may be triggered by proteins specific for bacteria through ‘molecular mimicry,’ which provide a hopeful direction for further study on the pathogenesis of AILDs. In short, the relationship between the organisms and AILDs needs to be explored.

## Conclusion

Increasing evidence has highlighted the crucial role of microbiome in AILDs. Research showed various changes of microbiome in metagenomic and metabolomic analyses. Although the studies so far didn’t clearly demonstrate the causation between microbiome and AILDs, they found the associated microbiome and even possible role in the pathogenesis providing direction for further study in the pathogenesis of AILDs. Moreover, mutual authentication among metagenomic and metabolomic of the microbiome could help us to understand the detailed and correct role of microbiota in AILDs. Nevertheless, the integrated analysis of gut microbiome and metabolite is lacking because of limited data of metagenomics and metabolomics in AILDs. With the application of metagenomics and metabolomics, it is possible to identify new microbial diagnostic markers in the early diagnosis and novel treatments of AILDs. The role of the microbiome, not only bacteria, in the mechanisms of AILDs needs more comprehensive and in-depth research to explore in the future.

## Author Contributions

BW and LZ guided the outline and carried out manuscript editing. YZ and YR collected data and drafted the manuscript. HZ performed manuscript review. Each of the co-authors has approved the final draft submitted. All authors contributed to the article and approved the submitted version.

## Conflict of Interest

The authors declare that the research was conducted in the absence of any commercial or financial relationships that could be construed as a potential conflict of interest.

## Publisher’s Note

All claims expressed in this article are solely those of the authors and do not necessarily represent those of their affiliated organizations, or those of the publisher, the editors and the reviewers. Any product that may be evaluated in this article, or claim that may be made by its manufacturer, is not guaranteed or endorsed by the publisher.
